# Effectiveness of an implementation strategy for a breastfeeding guideline in Primary Care: cluster randomised trial

**DOI:** 10.1186/1471-2296-12-144

**Published:** 2011-12-30

**Authors:** Susana Martín-Iglesias, Isabel del-Cura-González, Teresa Sanz-Cuesta, Celina Arana-Cañedo_Argüelles, Mercedes Rumayor-Zarzuelo, Marta Álvarez-de la Riva, Ana M Lloret-Sáez_Bravo, Rosa M Férnandez-Arroyo, José L Aréjula-Torres, Óscar Aguado-Arroyo, Francisco Góngora-Maldonado, Manuela García-Corraliza, Nazareth Sandoval-Encinas, Margarita Tomico-delRío, Ana M Cornejo-Gutiérrez

**Affiliations:** 1 Dirección Asistencial Sur Atención Primaria, Servicio Madrileño de Salud, (Avenida Juan de la Cierva, s/n), Getafe, (28902). Spain; 2 Unidad de Apoyo a la Investigación Gerencia Atención Primaria, Servicio Madrileño de Salud, (Calle Espronceda, 24), Madrid, (28003), Spain; 3 Centro de Salud Mª Ángeles López Gómez, Servicio Madrileño de Salud, (Calle María Ángeles López Gómez, 2), Leganés, (28915), Spain; 4 Hospital Clínico San Carlos, Servicio Madrileño de Salud, (Calle Profesor Martín Lagos, s/n), Madrid, (28040), Spain; 5 Centro de Salud Mendiguchía Carriche, Servicio Madrileño de Salud, (Plaza Comunidad de Madrid, s/n), Leganés, (28914), Spain; 6 Centro de Salud Huerta de los Frailes, Servicio Madrileño de Salud, (Avenida de los Pinos, 30), Leganés, (28914), Spain; 7 Centro de Salud Jaime Vera, Servicio Madrileño de Salud, (Avenida Europa, 1), Leganés, (28915), Spain; 8 Dirección Técnica de Sistemas de Información Gerencia Atención Primaria, Servicio Madrileño de Salud, (Calle O'Donnell, 55), Madrid, (28009), Spain; 9 Centro de Salud Francia, Servicio Madrileño de Salud, (Calle Francia, 38), Fuenlabrada, (28943), Spain; 10 Consultorio Local Moraleja de Enmedio, Servicio Madrileño de Salud, (Calle La Fuente, s/n), Moraleja de En medio, (28950), Spain; 11 Centro de Salud Alicante, Servicio Madrileño de Salud, (Calle Alicante, s/n), Fuenlabrada, (28945), Spain; 12 Centro de Salud Humanes, Servicio Madrileño de Salud, (Calle Ferrocarril, s/n), Humanes, (28970), Spain; 13 Hospital Universitario Ramón y Cajal, Servicio Madrileño de Salud, (Carretera de Colmenar Viejo, km. 9,100), Madrid, (28034), Spain

## Abstract

**Background:**

The protection and promotion of breastfeeding is considered a priority in Europe where only 22% of infants less than 6 months old are exclusively breastfed. In Spain this percentage reaches 24.8% but in our city it falls to 18.26%. Various studies emphasise that the improvement of these results should be based upon the training of health professionals. Following the recommendations of a breastfeeding guide can modify the practice of health professionals and improve results with respect to exclusively or predominatly breastfed children at 6 months of age.

**Method/Design:**

This study involves a community based cluster randomized trial in primary healthcare centres in Leganés (Madrid, Spain). The project aims to determine whether the use of an implementation strategy (including training session, information distribution, opinion leader) of a breastfeeding guideline in primary care is more effective than usual diffusion.

The number of patients required will be 240 (120 in each arm). It will be included all the mothers of infants born during the study period (6 months) who come to the health centre on the first visit of the child care programme and who give their consent to participate. The main outcome variable is the exclusive o predominant breastfeeding at 6 moths of age..

Main effectiveness will be analyzed by comparing the percentage of infants with exclusive or predominant breastfeeding at 6 months between the intervention group and the control group. All statistical tests will be performed with intention to treat. Logistic regression with random effects will be used to adjust for prognostic factors. Confounding factors or factors that might alter the effect recorded will be taken into account in this analysis.

**Discussion:**

Strategies need to be found which facilitate the giving of effective advice on breastfeeding by professionals and which provide support to women during the breastfeeding period. By applying the guide's recommendations, clinical variability can be reduced and the care received by patients can be improved.

**Trial registration:**

The trial was registered with ClinicalTrials.gov, number NCT01474096

## Background

### Current breastfeeding situation

The World Health Organization (WHO) considers breastfeeding the ideal way to feed infants, as it provides them with the nutrients needed for adequate growth and development, recommending exclusive breastfeeding until 6 months [1] and its continuance in supplemented form until 2 years of age [2].

Nowadays efforts are being made in both health and non-health areas for promoting the initiation and continuation of breastfeeding. Its benefits as much to the mother as to the infant, are widely known and it is thought to be an important factor in the promotion of children's health [3-6]. In spite of this, breastfeeding statistics are far from the levels considered adequate by the WHO, which estimates that 97% of women of fertile age are able to breastfeed their children.

In the latest *The State of the World's Children Report*, published in 2007 by the United Nations Children's Fund (UNICEF), it is noted that breastfeeding figures in Europe are discouraging (22% exclusive breastfeeding in infants less than 6 months old) [7]. In our country there is no official record system to make available data on the tracking and monitoring of breastfeeding. According to information from the latest *National Survey of Health*, in Spain breastfeeding at 6 months of age is 24.72% and in Madrid 23.54% [8]. In the study undertaken by the Spanish Paediatrics Association it is concluded that the average length of breastfeeding in Spain is about 3 months and only 24.8% of infants are breastfed at 6 months of age [9]. The data which has been obtained from existing records of computerised health reports in Primary Healthcare centres (PHCC) in our city show us a rather more unfavourable panorama as the percentage of 18.26%.

The WHO enumerated "*Ten Steps Towards Happy and Natural Breastfeeding" *[10] which are the basis for the introduction of projects based on the Baby Friendly Hospital Initiative (BFHI) which establishes an integrated approach to increase breastfeeding and has shown very positive results according to some studies [11,12].

### The role of health professionals

The protection and promotion of breastfeeding has been considered a priority in Europe, establishing the training of health professionals who must give such advice as one of the aspects upon which it should be based. The development of training systems which give professionals the knowledge, skills and attitudes required for the management of breastfeeding is considered necessary [13], since it has been seen the role of health professionals in breastfeeding rates [14].

Training in breastfeeding management is normally a shortfall in the undergraduate training of health professionals; this results in a lack of knowledge of the subject which has been made clear in many studies [15-17]. This shortfall in training persists even in the case of professionals with more specialist post-graduate training in this area. Thus, in the study undertaken by Temboury [15] on breastfeeding knowledge possessed by paediatric interns throughout Spain, using a self-completed questionnaire, it was concluded that there are important gaps in knowledge. It is noteworthy that the results differ from province to province, a fact which the researchers attribute to the different training plans and activities to improve breastfeeding which have been put into practice with institutional support in each province.

Akuse et al. [16] surveyed health and non-health professionals and found that although breastfeeding is thought of as highly important, a large percentage of health professionals tended to systematically recommend supplements for baby-feeding unnecessarily. The majority of the health professionals had received undergraduate training on the subject, but only a third had received specific training on the clinical approach to breastfeeding, something considered indispensable in clarifying such concepts.

In the study by Smale et al. [17], a qualitative investigation was undertaken with interviews and discussion groups. The results show that many health professionals do not feel able to give suitable support to breastfeeding mothers, consider that they receive little specific training in breastfeeding and feel that what they do receive refers above all to the anatomy, physiology and benefits of breastfeeding, but not to the tools needed to tackle the problems which can present themselves during breastfeeding.

Support by professionals has beneficial effects on the length of the breastfeeding, and the setting up of training projects has resulted in the improvement of breastfeeding indicators in the places where they have been developed. This confirms the fact that the training of professionals is closely linked to the commencement and continuance of breastfeeding. Travers et al. [18] found that some practices and opinions of paediatricians and nurses, such as not considering important the recommendations with regard to the duration of breastfeeding or the giving of adapted formula supplements to infants showing no gain in weight, were related to the probability of exclusive breastfeeding being continued or not.

For all these reasons the education and continuing training of health professionals working in Primary Care so that they acquire the necessary competence to suitably orientate mothers is a priority [19].

There are numerous strategies which can be employed on health professionals in order to encourage the promotion of breastfeeding. In a revision by Cochrane which evaluates the effectiveness of different breastfeeding support schemes [20], it is concluded that additional professional support is effective in prolonging breastfeeding and that the training courses for professionals recommended by the WHO are an efficient strategy for training health professionals. In a study undertaken in the Malaga Hospital [21], in which training courses for professionals as well as other community activities to promote breastfeeding were carried out, an increase took place in the length of breastfeeding in mothers who had given birth by caesarean section. In addition, the WHO and UNICEF have proposed the use of some training techniques to improve this situation [22]. They state the need for health professionals to have specific training on breastfeeding counselling in courses between 20 and 40 hours in length and, in the same way, health service administrators should attend 12-hour courses, with the intention of correcting the gaps in knowledge which currently exist.

### Guideline implementation strategies

In Madrid in the public health system, the Child Care Programme. (0-14 years old) is carried out in Primary Care. Within the framework of this programme, periodic visits are made to the nursing clinic and/or to the paediatrician for health examinations or for health education. In our city, taking into account the low level of exclusive breastfeeding at 6 months of age, the efforts which are made by the programme to promote breastfeeding are thought to be insufficient. For this reason it has been developed a breastfeeding action guide in coordination between Primary Care and the maternity hospital.

The adoption by health professionals of the recommendations included in the guide and their application to their patients means a process of change in clinical practice and, as such, is complex and depends on multiple factors [23]. It is essential that professionals involved participate both in their distribution as well as in the implementation strategies that are contemplated.

Whatever the implementation strategy, it should have an impact at four levels: increasing the knowledge of health workers and patients; changing attitudes, habits and behaviour of professionals in their clinical practice; taking into account also patients' preferences and administrative and economic influences; and modifying results, which signifies improving the quality of medical assistance and finally the health of the population by compliance with the recommendations of the Practice Guideline [24,25].

There exist various strategies for the implementation of practice guidelines which have shown their efficiency.

The Cochrane group EPOC (Effective Practice and Organization of Care) suggests they be classified in four categories: professional interventions (such as distribution of educational materials or educational meetings); financial interventions (such as fee-for-service or prospective payment); organisational interventions (such as creation of clinical multidisciplinary teams); and regulatory interventions (any intervention that aims to change health services delivery or costs by regulation or law) (EPOC Data Collection Checklist 2009) [26].

The revision by Grimshaw et al. [27] evaluates the effectiveness and cost of different organizational strategies for the dissemination-implementation of practice guidelines. It stands out that the majority of implementation strategies improve adherence to the practice guidelines. Simple strategies like checklists increase adherence by 14.1%. The distribution of educational materials increases it by 8.1%. Educational programmes, almost always as components of more complex interventions, improve practice by 6%, and audit inspections and feedback by 7%. In their conclusions it is pointed out that there is scarce evidence on which strategies are the most effective in each situation, for which reason the authors consider necessary the development and validation of theoretical models of behaviour change, as well as investigation of the efficiency of the strategies given the presence of different obstacles and factors which may modify effectiveness.

Without doubt, to achieve this change in health professionals demands that intervention strategies be realistic and adapted to each context. We consider essential the introduction of organizational strategies which allow us to generalise the use of the guide and the application of its recommendations as well as to measure and evaluate the impact which the implementation of the practice guideline may have both on professionals, modifying their practice, and on patients, improving their health results [28,29].

Our investigation proposal is based on the premise that we will be able to reach all health professionals involved in the care of mothers and infants by doing the training on site in their Primary Healthcare Centre (PHCC). By evaluating the effectiveness of this type of intervention we will be able to produce rigorous continuous training plans and, even more importantly, be able to know the results for the patient. For all these reasons we think it essential to know which strategies are the most suitable for the integration to take place in an effective way. Because we consider inevitable a 'contamination' effect of influence on each other between professionals working in the same centre, a cluster design has been chosen so that the interventions which do or do not take place will include all the professionals in each PHCC.

## Hypothesis

The implementation of a breastfeeding guideline, with the additional support of training and information directed at the professionals who impart the care, implies a greater integration of the guide's recommendations in usual clinical practice. As a result of this integration, an increase in the percentage of breastfed infants and a greater duration of breastfeeding will be produced.

The proportion of infants using exclusive breastfeeding (EBF) or predominant breastfeeding (PBF) at six months will increase at least 20% with respect to the control group (38% for the intervention group compared with 18% for the control group).

## Objectives

The aim of the present work is to determine whether an implementation strategy for a breastfeeding guide is more effective than the usual diffusion in terms of increasing the percentage of infants using EBF or PBF at 6 months.

Secondary objectives:

To determine whether an implementation strategy for a breastfeeding guide is more effective than the usual diffusion in terms of increasing the percentage of infants using EBF or PBF at 1, 3 and 12 months.

To determine whether an implementation strategy for a breastfeeding guide is more effective than the usual diffusion on duration of EBF and PBF.

To evaluate knowledge on breastfeeding possessed by health professionals prior to undertaking the intervention.

Describe the reasons for withdrawal from breastfeeding in both groups.

## Methods/Design

### Design of the study

This study is a community, parallel clinical trial, randomised by clusters, that compares two different guide implementation strategies. The intervention will be carried out on health professionals (doctors and nurses) in the PHCCs in Leganés, Madrid (Spain). The town of Leganés has nine PHCCs. for a population of 182471 inhabitants

The randomization units will be the PHCCs (clusters). The units of analysis are the mother-infant pair attended by the Leganés PHCCs. This design by clusters minimises possible contamination effects between centres. (check list cluster consort in additional file 1)

The study protocol was approved by the Area 9 Research Ethics Committee (2008/12/01), and met all good clinical practice demands.

### Subjects of the study

Mother of infants born between 1st June 2010 and 1st December 2010 who came to the PHCC on the first visit of the Child Care Programme.

#### Inclusion criteria

a. Be able to meet the demands of the trial and to be localisable for the next year, and to have the capacity to understand the questionnaires presented.

b. Give signed informed consent to be included in the study, and meet no exclusion criteria.

#### Exclusion criteria

Mothers and infants with contraindications for breastfeeding will be excluded:

a. Mothers' contraindications: HIV positive, substance abuse, chemotherapy, radioactive isotope treatments until the elimination of the isotope from the mother's body, active tuberculosis, active chickenpox, active Herpes lesions, Chagas disease.

b. Infants' contraindications: galactosemia.

### Sample size

For an alpha of 0.05, a power of 80%, and in order to detect an increase of 20% in the percentage of exclusive or preferential breastfeeding at 6 months of age in the intervention group, the overall sample size required is 176 patients (88 in each arm of the study).

Since randomisation is by clusters, the sample size has to be larger than if simple randomisation is performed, in order to take into account the design effect. The intra-class correlation coefficient is 0.01 [30] and a mean cluster size is assumed to be 30 patients. The design effect is 1.29. Given these assumptions, and expecting a 5% loss rate at one year, the final sample size required is 240 (120 patients in each arm).

### Randomisation

The randomisation unit will be the PCHC. An independent statistician will randomly assign the 9 PHCCs to either an intervention group or a control group following a simple, computer-generated random sequence (Epidat 3.1 software).

Consecutive patients will be chosen to minimise the risk of bias in their selection. During consultations, patients will be informed about the study and asked whether they would like to take part. Those who accept will be asked to give their signed consent, and checks will be made to ensure they meet all inclusion criteria but no exclusion criteria.

### Masking

In a study of this type it is impossible to mask the intervention. The analysis data will be performed by independent professionals blinded to the assignment group.

### Intervention

#### Control group (usual diffusion)

Presentation of the Breastfeeding Guideline in a one-off centralised clinical session for all PHCCs, 60 minutes in length. Two members of each of the 9 PHCCs will be invited to attend.

The Breastfeeding Guideline will be made available on the PHCC's webpage.

#### Intervention group

As well as the usual diffusion, the following implementation strategy will be carried out in each PHCC:

Training: three training sessions of one and a half hours in length will take place for all the health professionals. These will be given in the PHCC by the investigators.

Responsible person/opinion leader: a professional responsible for breastfeeding in each PHCC will be appointed (doctor, nurse, midwife or social worker).

### Variables

#### Outcome variables

The main outcome variable is the mother-infant pairs using EBF o PBF at six months

The secondary outcome variables will be:

##### Professional variables

Socio-demographic: sex, age, number of children.

Professional profile: paediatrician/midwife/nurse/nursing assistant/family doctor/medical intern (family/paediatrician). Years of professional experience. Breastfeeding training received and level of breastfeeding knowledge measured by means of Temboury's questionnaire [15].

##### Mother-infant pair variables

Mother: age, nationality, member of a stable couple, educational level (low - primary studies only; medium - high school completed, technical training or other non-university studies completed; high - university level education completed). Working situation of the mother: if she works outside the home and the number of hours per working day/days per week.

Prior information: If she has received breastfeeding information during her pregnancy and if she has attended prenatal training sessions.

Previous pregnancies: if affirmative, her pregnancy history will be recorded.

Previous breastfeeding experience (details for each child): length and type of breastfeeding for previous children; exclusive/preferential/partial. Reason for ceasing breastfeeding: mother's decision/no weight-gain/contraindication because of mother's illness (state which)/contraindication because of child's illness (state which)/contraindication with medications/nipple problems/other technical difficulty.

Current pregnancy: Birth date and type of birth: vaginal/caesarean; single/multiple. Gestational age of child in weeks, birth-weight in grams. Breastfed: a) if yes: EBF or PBF; b) if not: date of ceasing breastfeeding and the reason for abandoning breastfeeding.

### Data collection method

a) From professionals: the collection of socio-demographic data on the health professionals and the completion of the questionnaire on breastfeeding knowledge will take place before the start of any other activity.

b) From mothers: The inclusion of mothers in the study will take place on the infant's first consultation at the PHCC. The nurse and/or paediatrician will inform the mother and request her consent.

The collection of information about the breastfeeding will be by telephone interview, undertaken by an individual interviewer trained for the purpose; who does not know to which group the woman belongs.

Phonecalls will be scheduled at 1, 3, 6 and 12 months from the beginning of the study. During each call the mother will be asked if the infant is being breastfed at that time and, if the answer is negative, will be asked the date breastfeeding ceased and the reason for stopping. Each woman will be followed for a period of 12 months from the child's birth or until the time when breastfeeding ceases.

**Figure 1:**
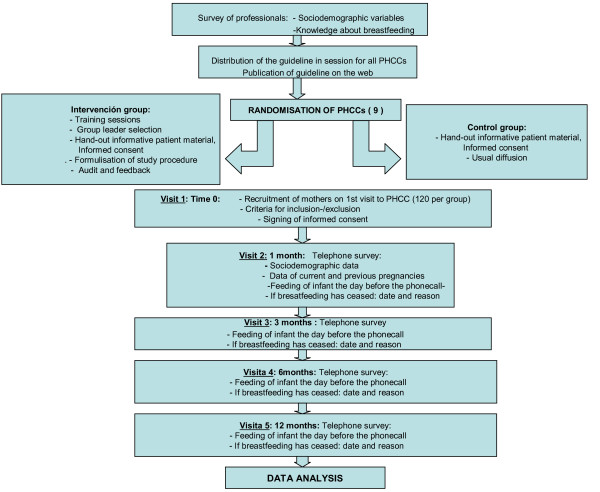
**Project structure**.

The timing of collection of data on the different variables is shown in Figure 1, in which the project structure and the data collection procedure are shown.

### Statistical Analysis

The use of randomisation by clusters conditions the statistical analysis that can be employed, especially in the calculation of confidence intervals and the testing of hypotheses. The following analyses will be undertaken:

Descriptive analysis of each variable with its corresponding CI at 95%. Description of the profile of patients who abandon the study plus their reason for withdrawal.

Comparison of the group at the beginning of the trial with regards to response variables, descriptive variables, and prediction factors. Bivariate statistical tests will be used suitable to the type of variable (qualitative or quantitative).

Analysis of primary outcome. There will be a comparison of the percentage of infants using EBF or PBF at 6 months in the two study arm using the Chi-squared test and the calculation of confidence intervals.

Logistic regression with random effects will be used to adjust for prognostic factors. Confounding factors or factors that might alter the effect recorded will be taken into account in this analysis.

To assess the effect of the intervention on the duration of the various types of breastfeeding, a survival analysis will be used comparing the two groups using the log-rank test. The control of potential confounding variables will be performed by the construction of various Cox regression models.

Effect of the intervention on secondary outcome variables: comparison of the proportion the percentage of EBF or PBF at 1, 3 and 12 months using the Chi-squared test and the calculation of confidence intervals.

All statistical tests will be performed with intention to treat. The last observation carried forward will be used for missing data. Significance will be set at P < 0.05. All calculations will be made using SPSS^© ^v.18 software.

### Ethical considerations

The study protocol was approved by the Research Ethics Committee on 1 December 2008 and met all good clinical practice demands right. This study has been funded by the Spanish Ministry of Science and Innovation via the Instituto de Salud Carlos III (PI08/90680).

## Discussion

Strategies need to be developed to confer on health professionals the knowledge, skills and attitudes necessary for breastfeeding management [13], given the repercussion which this has been seen to have had on breastfeeding levels [14]. In the framework of the National Health System and within the compass of Primary Care in our country, it is not always possible to establish training strategies of the duration and exhaustiveness proposed by the WHO for breastfeeding training.

Practice guidelines could be extremely useful in the continuous training of health professionals and as a support to decision taking during consultations. They are, without doubt, an additional tool to be used so that health professionals gain the competence needed to orientate mothers [19].

For guides to be able to change practice they must be used, and to this end they must be known and understood. The distribution of the guides and their correct use requires the development of suitable implementation strategies. Our implementation proposal allows us to involve all healthcare centre professionals concerned in the care of breastfeeding mothers and their children.

One of the limitations of our study is that a double blind design can not be applied to the projected intervention. However, we have planned a blind evaluation of the result.

Information which mothers may receive from sources outside the health system may influence them in their breastfeeding decision. We consider this situation to be similar for both the intervention group and the control group.

Our study's main contribution is to be able to evaluate which strategies permit or do not permit the integration of the recommendations of the Practice Guidelines in the normal practice of health professionals by measurement of the results in the population.

The trial was registered with ClinicalTrials.gov, number NCT01474096http://ClinicalTrials.gov.

## Competing interests

The authors declare that they have no competing interests.

## Authors' contributions

SMI conceived of the study and participated in its design and coordination. TSC, ICG, JAT y OAA participated in the design of the study. CAC, MAR, ALS, RFA, FGM, MGC, NSE y MTR carried out the guidelines. SMI, MRZ, ICG, TSC, ACG directed the writing of the manuscript. Contributions were made by the remaining authors. All authors have read and approved the final manuscript.

## Funding

This study was funded by the Spanish Ministry of Science and Innovation via the Instituto de Salud Carlos III (PI08/90680).

## List of Abbreviatons

EBF: Exclusive breastfeeding; PBF: Predominant breastfeeding; PHCC: Primary Healthcare Centre; UNICEF: United Nations Children's Fund; WHO: World Health Organization.

## Pre-publication history

The pre-publication history for this paper can be accessed here:

http://www.biomedcentral.com/1471-2296/12/144/prepub

## Supplementary Material

Additional file 1 **Cluster consort check list**. Review cluster consort check list. Click here for file
