# Supplementary material for: Effectiveness of an implementation strategy for a breastfeeding guideline in Primary Care: cluster randomised trial
Source: BMC Fam Pract. 2011 Dec 30;12:144. doi: 10.1186/1471-2296-12-144 (PMC3339325; doi:10.1186/1471-2296-12-144)
Supplement: Additional file 1 — Cluster consort check list. Review cluster consort check list. [file 1471-2296-12-144-S1.DOCX]

**Checklist of items to include when reporting a cluster randomised trial**

**Effectiveness of an implementation strategy for a breastfeeding guideline in Primary Care: cluster randomised trial**

| * = addition to CONSORT *Modifications to checklist in italics* | | | |
| --- | --- | --- | --- |
| PAPER SECTION  and topic | Item | Descriptor | Reported on Page No. |
| TITLE & ABSTRACT | 1* | How participants were allocated to interventions (e.g., “random allocation”, “randomised”, or “randomly assigned”), *specifying that allocation was based on clusters* | 1-2 |
| INTRODUCTION  Background | 2* | Scientific background and explanation of rationale, *including the rationale for using a cluster design.* | 2 - 6 |
| METHODS  Participants | 3* | Eligibility criteria for participants *and clusters* and the settings and locations where the data were collected. | 7 - 8 |
| Interventions | 4* | Precise details of the interventions intended for each group, *whether they pertain to the individual level, the cluster level or both,* and how and when they were actually administered. | 8-10  12 (Figure 1) |
| Objectives | 5* | Specific objectives and hypotheses, *and whether they pertain to the individual level, the cluster level or both*. | 6 - 7 |
| Outcomes | 6* | Report clearly defined primary and secondary outcome measures, *whether they pertain to the individual level, the cluster level or both*, and, when applicable, any methods used to enhance the quality of measurements (e.g., multiple observations, training of assessors). | 8 - 9 |
| Sample size | 7* | How *total* sample size was determined *(including method of calculation, number of clusters, cluster size, a coefficient of intracluster correlation (ICC or k), and an indication of its uncertainty*) and, when applicable, explanation of any interim analyses and stopping rules. | 7 - 8 |
| Randomisation.  Sequence generation | 8* | Method used to generate the random allocation sequence, including details of any restriction (e.g., blocking, stratification, *matching*). | 8 |
| Allocation concealment | 9* | Method used to implement the random allocation sequence, *specifying that allocation was based on clusters rather than individuals and* clarifying whether the sequence was concealed until interventions were assigned. | 8 |
| Implementation | 10 | Who generated the allocation sequence, who enrolled participants, and who assigned participants to their groups. | 8 |
| Blinding (Masking) | 11 | Whether or not participants, those administering the interventions, and those assessing the outcomes were blinded to group assignment. If done, how the success of blinding was evaluated. | 8 |
| Statistical methods | 12* | Statistical methods used to compare groups for primary outcome(s) *indicating how clustering was taken into account*; methods for additional analyses, such as subgroup analyses and adjusted analyses. | 10 |
| RESULTS  Participant flow | 13* | Flow of *clusters and* individual participants through each stage (a diagram is strongly recommended). Specifically, for each group report the numbers of *clusters and* participants randomly assigned, receiving intended treatment, completing the study protocol, and analyzed for the primary outcome. Describe protocol deviations from study as planned, together with reasons. | Not applicable (a) |
| Recruitment | 14 | Dates defining the periods of recruitment and follow-up. | Not applicable (a) |
| Baseline data | 15* | Baseline information for each group *for the individual and cluster levels as applicable* | Not applicable (a) |
| Numbers analyzed | 16* | Number of *clusters and* participants (denominator) in each group included in each analysis and whether the analysis was by “intention-to-treat”. State the results in absolute numbers when feasible (e.g., 10/20, not 50%). | Not applicable (a) |
| Outcomes and Estimation | 17* | For each primary and secondary outcome, a summary of results for each group measures *for the individual or cluster level as applicable*, and the estimated effect size and its precision (e.g., 95% confidence interval) *and a coefficient of intracluster correlation (ICC or k) for each primary outcome.* | Not applicable (a) |
| Ancillary analyses | 18 | Address multiplicity by reporting any other analyses performed, including subgroup analyses and adjusted analyses, indicating those pre-specified and those exploratory. | Not applicable (a) |
| Adverse events | 19 | All important adverse events or side effects in each intervention group. | Not applicable (a) |
| DISCUSSION  Interpretation | 20 | Interpretation of the results, taking into account study hypotheses, sources of potential bias or imprecision and the dangers associated with multiplicity of analyses and outcomes. | Not applicable (a) |
| Generalisability | 21* | Generalisability (external validity) *to individuals and/or clusters (as relevant)* of the trial findings. | Not applicable (a) |
| Overall evidence | 22 | General interpretation of the results in the context of current evidence. | Not applicable (a) |

(a) This is a protocol study, the items of results and discussions/interpretation are not applicable.
